# Dr. Muhammed Majeed: Father of Indian nutraceuticals industry- a life profile

**DOI:** 10.1016/j.jaim.2024.101074

**Published:** 2025-01-10

**Authors:** Anju Majeed, Shaheen Majeed, Amirhossein Sahebkar

**Affiliations:** a Sami-Sabinsa Group Limited, 19/1, 19/2, I Main, II Phase, Peenya Industrial Area Bangalore, Karnataka, 560 058, India; b Sabinsa Corporation, 750 Innovation Circle, Payson, UT, 84651, USA; c Center for Global Health Research, Saveetha Medical College and Hospitals, Saveetha Institute of Medical and Technical Sciences, Saveetha University, Chennai, India; d Biotechnology Research Center, Pharmaceutical Technology Institute, Mashhad University of Medical Sciences, Mashhad, Iran; e Applied Biomedical Research Center, Mashhad University of Medical Sciences, Mashhad, Iran

## 1 Introduction

**Figure:**
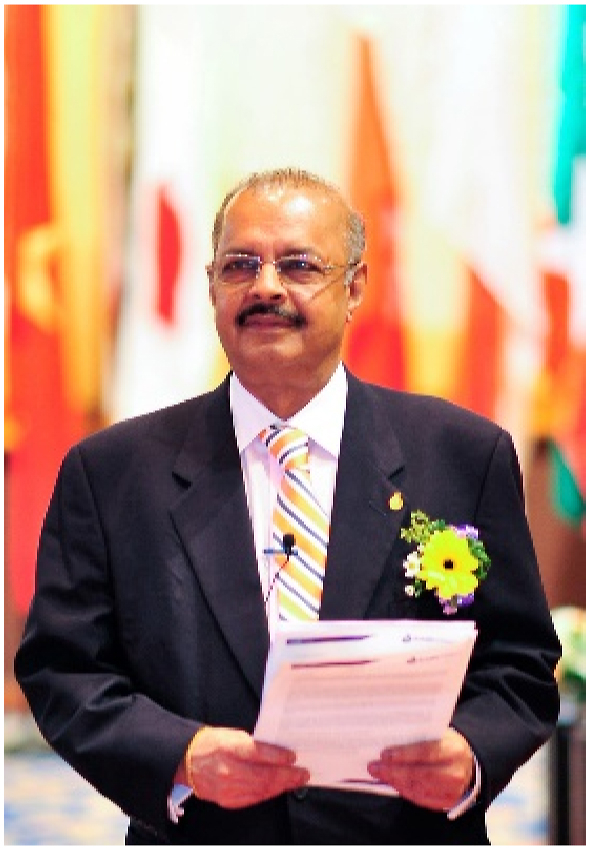

Dr. Muhammed Majeed, Dr. MM as he was addressed, was a scientist by heart. An incredibly well-groomed and cheerful person with excellent communication skills and a strong work ethic, he was a dedicated scientist. Dr. Majeed was the pioneer who created a global market for standardized herbal extracts, utilizing modern research to expand the knowledge of Ayurveda. Dr. Majeed encouraged his people to seek new scientific achievements without being too cautious. He would often say that we should dream with our eyes open. His passion for science enabled him to establish elaborate research and development units covering everything from chemistry to biology, including formulations and food science. The gleam in his eyes when he used to describe new scientific findings and proudly display the R&D facilities will be missed by those who worked with him. Dr Majeed believed that the new medicines and vaccines from pharmaceutical companies will not be affordable for most Indians. Hence, he wanted to create innovative products based on the Ayurvedic principles that prevent health problems and meet the needs of the time.

His many accomplishments are expounded upon in this biographical profile.

## 2 Early life

Dr Muhammed Majeed was born on September 25, 1948, in Kollam district of Kerala, India. He lost his father at the age of 12 after which his family faced difficulties and hardship. However, Majeed's mother, Fathima, ensured her son was well educated. After earning an undergraduate degree in Pharmacy from the Thiruvananthapuram Medical College in 1973, Dr. Majeed worked as a teacher before emigrating to the USA in 1975 with only $8 in his pocket. Initially working in a pharmacy, he pursued his master's in industrial pharmacy from Long Island University (New York, USA). Subsequently, in 1986, he earned his PhD in Industrial Pharmacy from St. John's University in New York, USA. He held head of research positions at Pfizer, Carter Wallace, and Paco Pharmaceuticals during his tenure in the pharmaceutical sector.

## 3 Dr Majeed – the entrepreneur

With the incorporation of Sabinsa Corporation, a research-oriented pharmaceutical and nutraceutical company in in New Jersey in 1988, the entrepreneur in Dr Majeed was born. His immense scientific knowledge and business acumen were his greatest investments and thus began an inspiring success story. In 1991, Dr. Majeed established Sami Labs Limited (now known as the Sami-Sabinsa Group Limited (SSGL), a research and manufacturing facility in Bengaluru, India. He expanded his business by acquiring America's Finest, Inc. (AFI), in 1992, a dietary supplement company specializing condition-specific formulations. He had to convince his customers, who thought of this acquisition as a competition. But Dr. Majeed felt it would help them understand how to deliver the raw materials in a finished formulation. Additionally, placing a finished formulation in the marketplace with the ingredients he invented meant consumers had immediate access and more choices for their health. He opened a new processing and distribution facility in Payson, Utah, in 1999, which was later expanded to support new product research, development, and contract manufacturing. To encourage the buying and selling of spices, Sami Labs established Sami Spices, a division, in Cochin, India, in the beginning of 1999. Organica Aromatics was introduced to cater to the flavor and scent sector in the same year. To sell specialty ingredients to the personal care and cosmetics sectors, Sabinsa Cosmetics was established in 2001. The Sami-Sabinsa Group built businesses in Australia, Brazil, Canada, China, Germany, Japan, Poland, South Africa, South Korea, UAE, and Vietnam over the course of 36 years, in addition to expanding its activities in the United States and India. The group also opened satellite offices in France, Hungary, Spain, Taiwan, and the United Kingdom. Early on, he noticed that regulatory requirements differed in each country, which could pose a hurdle in expanding the business across the globe. He used his resources to understand and overcome regulatory hurdles, again with the belief that his ingredients should reach every corner and consumers should have access to such healthful ingredients. He set up a dedicated technical support team that liaised with local country offices. The tech support team's primary objective was to ensure training for the sales staff worldwide and to help with regulatory documentation where needed. To spread the message about the ingredients he developed, he created a traveling seminar program called Sabinsa On Wheels (SOW). It was packed with scientific presentations that would be delivered in major cities worldwide.

Dr. Majeed was an embodiment of hard work with faith in scientific reasoning and strong conviction in Ayurveda. His conclusions were grounded in science, therefore when a customer requested bark from a tree, rather than crushing it and placing it in a capsule, he tried to comprehend what they were trying to do. In one such instance, a customer discovered a use in Ayurveda but could not isolate the particular bioactive compound that made a healthful benefit. That is where Dr. Majeed's pharmaceutical background came into play. He was able to create an extraction method that elucidated the key components from that bark, the principal actives that would give you the benefits that were written about traditionally. This bark is from the *Commiphora mukul* tree, and guggul is the component, which he branded and sold as Gugulipid®. For more than 35 years, Dr. Majeed developed each product using the same thought process. He believed that modern science should validate traditional knowledge.

Today, the Sami-Sabinsa Group Limited, having its corporate headquarters and a state-of-the-art R&D facility in Peenya, Bengaluru, India, employs over 1400 people globally. Focusing on specialized research and product development, the R&D team includes more than 120 senior scientists who dig into the mechanisms of action and physiological effects of herbs and share that knowledge through publication in peer-reviewed journals.

## 4 Novel products and patents

Dr Majeed firmly believed that patent protection was important in the natural product ingredients business. In his own words *"It was back in 1995 I learned a lesson the hard way. My first blockbuster product 'Garcinia' was copied and sold at extremely low prices by competitors in the U.S. market. That is the time that I learned that it is a patent that will protect you from people putting your brand name on their ingredients. That protection helps you* support *innovation with continued research."* Sami -Sabinsa group developed several branded, patented, and unique products which has helped establish the company's global identity. Dr. Majeed has over 480 U S. and internationally granted patents to his credit. Curcumin C3 Complex® is a patented composition of Curcuminoids from turmeric rhizomes which stands out from the crowded curcumin marketplace as being the "most extensively studied and clinically documented" Curcuminoids brand in the world today with more than 263 publications, including 109 clinical studies. BioPerine®, a standardized extract of black pepper is another innovation of Dr. Majeed. It has been recognized as the world's safest and most clinically studied extract standardized for 95% piperine, with 63 studies supporting its bioavailability-enhancing properties and other health benefits [1].

Dr. Majeed influenced the world's probiotic industry with a novel strain of heat-stable spore forming *Heyndrickxia coagulans* (*Bacillus coagulans)* MTCC 5856, named LactoSpore® [2]. It is generally recognized as safe (GRAS) by the U.S. Food and Drug Administration (FDA) and approved by Natural Health Product in Canada and by regulatory authorities in Australia, Europe and New Zealand.

Dr. Majeed introduced a range of branded products, such as ForsLean®, LeanGard®, Citrin®, GarCitrin® Shagandha®, SeleniumSeLECT®, Nigellin® and Sabroxy® which have provided global recognition for SSGL. Selenium SeLECT®, the company's branded L- (+)-Selenomethionine compound, the most bioavailable, pure, and safe form of selenium, also has self-affirmed GRAS status. The worldwide quality standard or United States Pharmacopeia (USP) monograph for selenomethionine is based on Selenium SeLECT®. In 1994, Selenium SeLECT® received the National Award for Quality and Innovation (Basic Drugs) from (Late) Dr. Shankar Dayal Sharma, President of India.

The Sami-Sabinsa Group is committed to providing innovative, patented nutraceutical and cosmeceutical products for its customers.

## 5 When quality meets sustainability- vision to provision

Dr. Majeed believed that a good supplement begins with quality ingredients supplied sustainably. At the same time, he believed any sustainability program must also assist the communities, improving their standard of living without compromising their sustainable way of life. He grew up with his father owning and working at a banana plantation, which later encouraged him to take on agricultural practices to ensure stable supply operations, which led to a better quality of life for thousands of farmers and their families. During his professional life, this would be instrumental for the further developments made at his companies worldwide; without a proper supply of natural materials, the business would suffer. He took this challenge on personally, visiting the farmers and municipalities to help set up buyback agreements and provide basic necessities for farmers to perform their functions. He identified small and marginal farmers in Salem and surrounding districts in the state of Tamil Nadu, India, to cultivate key Ayurvedic herbs. In 2006, Dr. Majeed gave these farmers and their communities planting supplies, training, and continuing assistance. He ventured into contract farming of medicinal plants and developed 40,000 acres of land in India dedicated for sustainable supply of quality raw materials. He established a unique contract cultivation model where the farmers are considered as partners. The returns to the farmers are maximized by assuring them an optimum price and the latest technical knowledge to improve their productivity. The farmers are always encouraged to follow Good Agricultural Practices (GAP) to ensure the quality of raw materials. He extended the contract cultivation model to Southeast Asian countries and Africa also. His company is committed to fair trade practices, and its contracts call for paying a guaranteed price even if the crop fails due to weather extremes and paying more for a harvest when market prices dictate.

Dr. Majeed and his company were also involved in an ambitious 10-year reforestation program of *Pterocarpus marsupium* (Indian Kino) trees in Madhya Pradesh, India and *Picrorhiza kurroa* in the Himalayas as an initiative to conserve highly valued but threatened tree species in India. *Coleus foskohlii* cultivation is another example of Sami-Sabinsa's largest and most successful contract cultivation programs. He motivated about 5000 farmers at Attur, a town in Tamil Nadu, to cultivate Coleus, which has now spread over more than 10,000 acres in different states of India. In fact, Dr. Majeed's efforts in bringing Coleus from the wild into planned cultivation have made the 10.13039/501100011700National Medicinal Plants Board of India to include Coleus in their list of ‘Prioritized Plants for Cultivation’ and grant farmers a 30% subsidy. The program has further helped many marginal farmers to switch to alternate crops and earn better margins.

## 6 Unveiling of humanity over commercial success

The charitable arm of the SSGL, the Dr. Majeed Foundation, provides educational help to underprivileged children. It gives women the opportunity to play a vital role in the socio-economic growth of the world. He started two training centers in India, one in Kochi, Kerala, and one in Kunigal, Karnataka, to educate MS Office and desktop application software to underprivileged children. Dr. Majeed foundation also sponsors meal plans for elderly charitable shelters and homes. The foundation has made significant financial contributions to State and Central Government aid programs for the management of natural disasters. Dr. Majeed Foundation distributed food kits to over 9000 beneficiaries in the States of Karnataka and Telangana in India to support the underprivileged whose means of subsistence were adversely affected by the pandemic lockdowns He also encouraged research in medicinal plants by giving research grants and awards for outstanding research in natural plant molecules, biotechnology, and biodiversity.

## 7 Setting an example and illuminating the path

Dr. Majeed was a multi-talented personality who strongly believed in consistent effort and hard work. In addition to being a scholar and businessperson, he had a strong sense of social responsibility. He ensured that all business decisions were made with the economic, social, ethical, and environmental improvement of society. Sami-Sabinsa Group Limited is built on his passion for high-quality research and is well-regarded by the scientific community domestically and internationally for its products. Finally, Dr. Majeed has greatly benefited scientists and businesses worldwide and has created an untold number of entrepreneurs who have followed in his footsteps. In an interview with Wholefood magazine in 2022, Dr Majeed said *"I believe if you bring ordinary people into leadership positions and give them the opportunity to fail, they will make much better leaders of the future. If you don't make mistakes, you are not doing anything."*

Dr. Majeed, a visionary, will be remembered for generations for his remarkable contributions to modern Ayurveda. His legacy will continue to make Sami-Sabinsa Group, a global leader in ingredient innovation, fulfilling his vision. He is survived by his daughters Anju and Sami, his son Shaheen, and grandchildren Rizwan, Johara, Nasneen, Ramses, Sunayna, Rajah, and Maximo.

## 8 Quotes about Dr. Majeed by benefited scientists and entrepreneurs

“I first met Dr. Majeed in 1999 in New Jersey at the headquarters of Sabinsa Corporation and I was very impressed by his clearness in our discussion and how he built up his company based on science and his ideas. I have very good memories of our business trips that we took together. His great support and trust in me when I started my own company as a partner of Sabinsa is something I will never forget in my life. To me, Dr. Majeed was an outstanding personality, entrepreneur, and a friend." *—Dr.Jürgen Liedek/MIVALIED.* [3]

“Dr. Majeed was one of the smartest and most fascinating men I have known. When I was first starting in the industry, so was he in the U.S. Their office was close to ours, and we would meet to put together small ads, and he would educate on Ayurveda and more. In 2017 I had the privilege to go to India and spend some time with Dr. Majeed. I treasure that time we had."*—Heather Wainer,* ***WholeFoods*** *Magazine Publisher, and VP of Media.*[3]

“Dr Majeed was a visionary in terms of the importance of both scientific substantiation and intellectual property in the nutraceutical ingredient space. While we connected many times through the years, I had the opportunity to spend time with Dr Majeed in India during the celebration of Sabinsa's 30-year anniversary. He was passionate about applying modern thinking and science to traditional Ayurvedic ingredients and making them available for human health worldwide. I'm hopeful that Sabinsa will continue to be a global leader in ingredient innovation, fulfilling Dr Majeed's vision." *—Heather Granato, President, Nutrachievement.* [3]

Mark Blumenthal, founder, and executive director of the American Botanical Council (ABC): India has been a treasure trove of medicinal plants and spices for millennia. Dr. Majeed was a key pioneer who helped modernize and develop the Indian Ayurvedic and medicinal plant tradition to a point where clinically tested, pharmaceutically prepared standardized botanical extracts have become a major export item for the Indian economy, providing jobs for thousands of people. He and Sabinsa were the first to expose the marketing of synthetic curcuminoids as additives to “standardized turmeric extract” and prevailed in litigation against sellers of this fraudulent material. Dr. Majeed's strong commitment to scientific and human clinical research on his company's botanical ingredients is the reason why Sabinsa was the first Indian company to receive ABC's Varro E. Tyler Award in 2023. [4]

## 9 Suzanne shelton, founder of The Shelton Group and longtime sabinsa publicist

"Sabinsa was one of my first clients after I started The Shelton Group in 1990. In the early years, I worked directly with Dr. Majeed. He was truly a visionary and committed to confirming, with modern research, the significant health benefits of traditional Ayurvedic herbs. Back then, publishing research on branded ingredients was rare, although competitors' “borrowing” of Sabinsa's science happened all too often, much to Dr. Majeed's distress. He never gave ground when protecting the products that he and his scientists developed. In his view, safeguarding Sabinsa's intellectual property was protecting its customers as well. It was fascinating to watch Dr. Majeed's leadership in digging deeply into an herb or herbal product, such as with curcumin and the multiple variations of that material Sabinsa created. Over the years, he pioneered many herbal products that are industry staples now. While Dr. Majeed could be tough, he was also a dedicated mentor when he saw potential in someone and personally stayed in touch with many of his employees for years. It is an honor to have been working for so many decades with the company he created and a great source of professional pride and satisfaction to have been part of Dr. Majeed's team." [4]

## 10 Awards and achievements

❖ Thomas Alva Edison Patent Award two times, 2004, 2005
❖ National Award for Quality and Innovation by the President of India in 1995, 1997, and 2008
❖ Department of Scientific & Industrial Research award in 2002
❖ Ellis Island Medal of Honor for Distinguished Americans in 2004
❖ Induction to the New Hope Network Hall of Legends in 2016
❖ Shield of Honor by The Associated Chambers of Commerce and Industry of India (ASSOCHAM) National Symposium in 2018
❖ The American Herbal Products Association's Herbal Industry Leader Award
❖ The American Botanical Council's Varro E. Tyler Commercial Investment in Phytomedicinal Research award
❖ Nutra Champion in the NutraIngredients Awards organized in association with VitaFoods Europe in 2018
❖ Indian Nutraceuticals Trailblazer award 2019
❖ Iconic and inspiring Leader of the nutraceutical industry award by Tomes of India in 2020
❖ Lifetime achievement award by ASSOCHAM 2020
❖ Legend of Natural Cosmeceuticals' award in 2020
❖ The Zandu International Oration Award for ‘Excellence in the field of Biology and research on Ayurvedic products’ by Indian Association for The Study of Traditional Asian Medicine (IASTAM) in 2020.
❖ Father of the Indian Nutraceuticals Industry by ASSOCHAM in 2022
❖ Legend of the Millennium in Nutraceutical Industry in 2022
❖ Lifetime Achievement Award (Posthumous 2024)

## Funding sources

This manuscript did not receive any external funding.

## Declaration of generative AI in scientific writing

No generative AI was used for drafting the manuscript.

## Author contributions

Conceptualization: AS; Writing-original draft: AS, Writing-review and editing: SM and AM.

## Data statement

The MS has no data for sharing.

**Figure:**
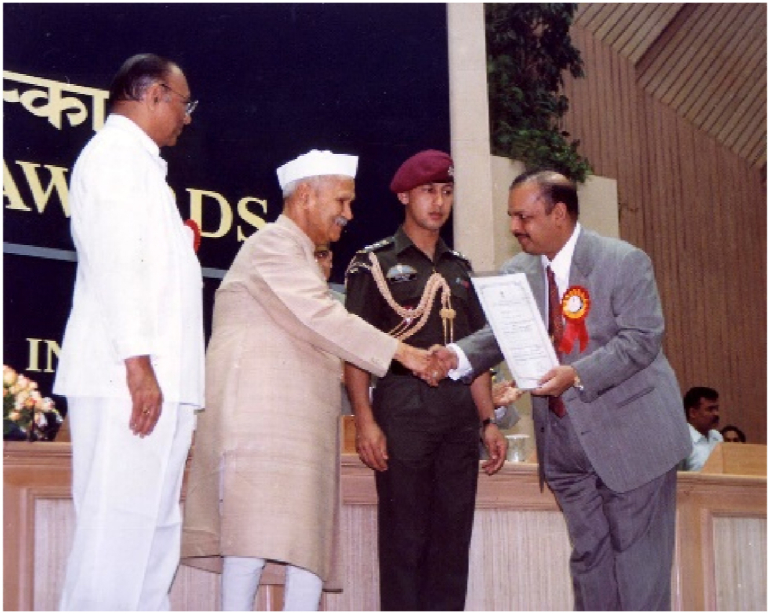
Dr. Majeed receiving the national award for Selenium SeLECT® from the president of India.

## Conflict of interest

The authors declare the following financial interests/personal relationships which may be considered as potential competing interests:Anju Majeed is the Group Executive Chairperson of Sami-Sabinsa Group and the daughter of Late Dr Muhammed Majeed. Shaheen Majeed is the Global Managing Director and Chief Executive Officer of Sami-Sabinsa Group and the son of Late Dr. Muhammed Majeed.
